# Earthquake Nucleation Along Faults With Heterogeneous Weakening Rate

**DOI:** 10.1029/2021GL094901

**Published:** 2021-11-09

**Authors:** Mathias Lebihain, Thibault Roch, Marie Violay, Jean‐François Molinari

**Affiliations:** ^1^ Laboratory of Experimental Rock Mechanics Civil Engineering Institute École Polytechnique Fédérale de Lausanne Lausanne Switzerland; ^2^ Laboratoire Navier École des Ponts ParisTech Université Gustave Eiffel CNRS (UMR 8205) Marne‐la‐Vallée France; ^3^ Computational Solid Mechanics Laboratory Civil Engineering Institute Materials Science and Engineering Institute École Polytechnique Fédérale de Lausanne Lausanne Switzerland

**Keywords:** Earthquake dynamics, friction, nucleation, homogenization, stability analysis

## Abstract

The transition from quasistatic slip growth to dynamic rupture propagation constitutes one possible scenario to describe earthquake nucleation. If this transition is rather well understood for homogeneous faults, how the friction properties of *multiscale asperities* may influence the *overall stability* of seismogenic faults remains largely unclear. Combining classical nucleation theory and concepts borrowed from condensed matter physics, we propose a comprehensive analytical framework that predicts the influence of heterogeneities of weakening rate on the nucleation length $L_{c}$ for linearly slip‐dependent friction laws. Model predictions are compared to nucleation lengths measured from 2D dynamic simulations of earthquake nucleation along heterogeneous faults. Our results show that the interplay between frictional properties and the asperity size gives birth to three instability regimes (local, extremal, and homogenized), each related to different nucleation scenarios, and that the influence of heterogeneities at a scale far lower than the nucleation length can be averaged.

## Key Points

- The nucleation length of heterogeneous faults with multiscale asperities of weakening rate can be predicted for slip‐dependent friction
- Our theory accounts for the transition in fault stability regimes from the traditional "weakest defect" theory to a homogenized behavior
- Only asperities larger than the nucleation length participate in the fault stability, while the influence of small asperities is averaged

## 1 Introduction

Understanding how interfaces fail is of utmost importance in fields ranging from earthquake physics to engineering fracture mechanics. For unstable frictional interfaces such as seismogenic faults, field observations (Bouchon et al., 2013; Kato et al., 2012) as well as laboratory experiments (Ben‐David & Fineberg, 2011; Dieterich, 1978; Latour et al., 2013; McLaskey, 2019; Ohnaka & Kuwahara, 1990) suggest one possible scenario where the onset of fault motion is characterized by the transition from quasistatic slip growth to dynamic rupture propagation (Passelègue et al., 2016; Svetlizky et al., 2016). The transition happens when a region of critical size $L_{c}$ of the fault is slipping. The knowledge of this *nucleation length* proves crucial since it allows to predict both the *loading levels* and the *position* at which the earthquake motion starts (Albertini et al., 2020; Ampuero et al., 2006; Uenishi & Rice, 2003).

Previous theoretical works linked $L_{c}$ to the frictional properties of the fault for linear slip‐dependent (Campillo & Ionescu, 1997; Dascalu et al., 2000; Uenishi & Rice, 2003) and more complex rate‐and‐state (Aldam et al., 2017; Brener et al., 2018; Ruina, 1983; Rubin & Ampuero, 2005; Viesca, 2016b) friction laws along *homogeneous* faults. Yet, frictional properties are expected to significantly vary along the fault plane and with depth due to changes in the local host rock lithology, roughness, or in situ conditions (normal stress, temperature, pore fluid pressure, etc.) (Ohnaka, 2003; Tse & Rice, 1986). Then, how do these *multiscale heterogeneous frictional asperities* influence the *global stability* of seismogenic faults? Recent studies (Albertini et al., 2020; de Geus et al., 2019; Dublanchet, 2018; Perfettini et al., 2003; Ray & Viesca, 2017, 2019) provide valuable insights on how heterogeneities impact the overall stability of frictional interfaces, but arguably oversimplify the complexity of natural faults by assuming either a homogeneous weakening rate (Albertini et al., 2020) or orderly placed asperities of uniform size (Dublanchet, 2018; Perfettini et al., 2003; Ray & Viesca, 2017, 2019). A comprehensive framework, which links the variations of frictional properties at all scales to the overall fault stability, is thus dearly lacking.

In this Letter, we build on the theory of static friction (Rubin & Ampuero, 2005; Uenishi & Rice, 2003; Viesca, 2016a) and the physics of depinning (Cao et al., 2018; Démery et al., 2014; Tanguy & Vettorel, 2004) to develop a theoretical framework that predicts, for *any* heterogeneous linearly slip‐dependent fault interface, the critical size $L_{c}$ of the earthquake nucleus. Supported by numerical full‐field dynamic calculations, we show that the nucleation of an earthquake is not only always triggered by the *weakest* heterogeneity, but can also emerge from the *collective* depinning of multiple asperities. We highlight that this shift in instability regime stems from the interplay between the characteristic size of the heterogeneity and the length scale set by the distribution of frictional properties. Finally, we show that, in assessing the stability of an interface, one has to mainly account for perturbations whose wavelength exceeds the nucleation length, since the influence of small‐scale asperities can be averaged.

## 2 Materials and Methods

### 2.1 Dynamic Simulations of Earthquake Nucleation Along Heterogeneous Faults

**Figure 1:**
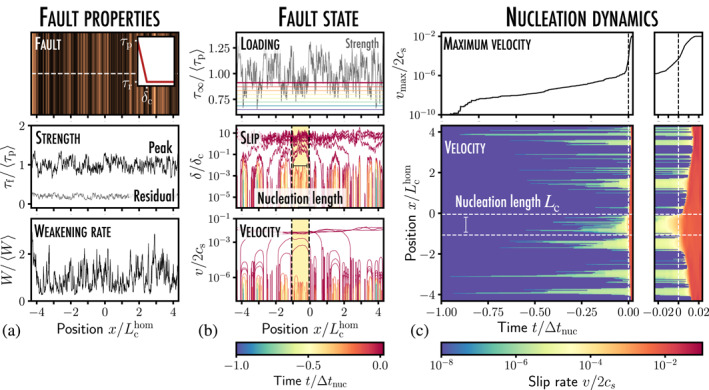
(a) Nucleation dynamics along a 1D heterogeneous coplanar fault constituted of brittle (in orange) and ductile (in black) asperities; inset: the fault frictional properties locally follow a linear slip‐dependent law; the frictional stress $\tau_{f}$ of the interface goes from its peak value $\tau_{p}$ to the residual one $\tau_{r}$ when the local slip δ reaches its critical value $\delta_{c}$, defining a weakening rate $W=(\tau_{p}-\tau_{r})/\delta_{c}$. $\tau_{p}$, $\tau_{r}$, and W are varying independently along the fault position x. (b) The heterogeneous fault is subjected to a uniform shear loading $\tau_{\infty }$. Under the influence of the steadily increasing loading, several regions of the fault start slipping where $\tau_{\infty }$ locally exceeds the strength $\tau_{p}$. (c) Slip growth develops quasistatically without any significant velocity burst, until one slip patch reaches a critical length $L_{c}$ that leads to the dynamic rupture of the whole interface (see Movie S1).

We consider two *homogeneous* 2D semi‐infinite elastic bodies that are kept in contact with a uniform normal pressure $\sigma_{n}$, idealizing the fault structure as a *planar* 1D frictional interface indexed by x. The fault is loaded through a macroscopic shear stress $\tau_{\infty }(x,t)$ that slowly increases in time t. The friction $\tau_{f}$ that opposes interface motion is assumed to be linearly slip dependent and fluctuates along the fault (Figure 1a). It locally evolves as slip grows from its peak value $\tau_{p}(x)$ to its residual one $\tau_{r}(x)$ with a weakening rate W(x) that describes the material brittleness/ductility. Variations of the frictional properties $\left(\tau_{p},\tau_{r},W\right)$ may emerge along natural faults due to local changes in geometry, roughness, lithology, or ambient conditions (Ohnaka, 2003; Tse & Rice, 1986). Recent works show that the nucleation along homogeneous (Viesca, 2016a) and heterogeneous (Ray & Viesca, 2017) faults in the (aging) rate‐and‐state framework could be investigated from the stability of an *equivalent* interface with *spatially dependent piecewise linear* slip‐weakening friction. Despite restrictive assumptions, our work may then provide ways to predict rupture nucleation for more complex and experimentally supported friction laws.

As the macroscopic loading $\tau_{\infty }(x,t)$ grows, it locally exceeds the friction $\tau_{p}(x)$, and the two bodies detach from one another by a slip δ(x,t) (Figure 1b). Provided that the fault has been at rest for a time far larger than that set by the propagation of elastic waves, the evolution of δ is described by the quasi‐dynamic equations of elasticity for Mode II cracks (Lapusta et al., 2000; Rice, 1993):

(1) $$\tau_{\infty }(x,t)-\frac{\mu^{*}}{2c_{s}}\frac{\partial \delta }{\partial t}(x,t)-\mu^{*}L[\delta ](x,t)=\max[\tau_{p}(x)-W(x)\delta (x,t),\tau_{r}(x)]$$

where $\tau_{\infty }$ is the far‐field macroscopic loading, $c_{s}$ the shear wave velocity, $\mu^{*}=\mu /(1-\nu )$ (μ and ν being the shear modulus and the Poisson's ratio, respectively), and $L[\delta ](x,t)=\frac{1}{2\pi }\int_{-\infty }^{+\infty }\frac{\partial \delta /\partial x^{\prime }(x^{\prime },t)}{\left(x-x^{\prime }\right)}dx^{\prime }$ is a linear operator. In Equation 1, the term $-\frac{\mu^{*}}{2c_{s}}\frac{\partial \delta }{\partial t}$, often called “*radiation damping*,” physically represents wave radiation from the interface to the two elastic bodies, while $\mu^{*}L[\delta ]$ represents the nonlocal contributions of the overall slip to the local stress state. To investigate the stability of such a heterogeneous fault, we run periodic dynamic simulations building on a spectral boundary integral formulation of fracture (Breitenfeld & Geubelle, 1998; Geubelle & Rice, 1995). These simulations account for both the static redistribution of stress of Equation 1 and dynamic stress transfers (see Section 1.2 in Supporting Information S1).

How is the fault stability influenced by the steadily increasing loading? It results in rather complex dynamics as can be observed in Figure 1b. Multiple regions slipping at an accelerated rate, referred to as “*slip patches*,” start to nucleate on the positions where $\tau_{p}$ is low. As the loading is further increased, they grow quasistatically and coalesce into larger slipping regions. This initial nucleation stage of duration $\Delta t_{\text{nuc}}$ proves rather quiescent since no major velocity burst is observed. Yet, at t=0, an instability develops on the right part the fault: a rupture propagates dynamically, and the two bodies start sliding one onto another at a uniform slip rate.

If such a simulation constitutes one realistic scenario for natural earthquakes nucleation, the simultaneous growth of multiple slip patches prevents any accurate measurement of the size $L_{c}$ of the instability nucleus, which might well be twice as large as our measurement of Figure 1c. Yet, identifying this critical length scale proves crucial since it gives access to (a) the loading levels (Uenishi & Rice, 2003) and (b) the position at which an earthquake nucleates (Albertini et al., 2020; Ampuero et al., 2006) when W is homogeneous along the fault.

These difficulties arise from spatial variations of peak strength that have been proven to play no role in the stability behavior of a slip‐dependent frictional interface, which is solely controlled by the weakening rate W (Favreau et al., 1999; Uenishi & Rice, 2003). Indeed, assuming that the macroscopic loading $\tau_{\infty }$ slowly increases enough and that the slip perturbation is small enough, the interface velocity $v=\dot{\delta }$ is described near the instability by Uenishi and Rice 2003:

(2) $$\frac{\mu^{*}}{2c_{s}}\frac{\partial v}{\partial t}(x,t)+\mu^{*}L[v](x,t)-W(x)v(x,t)=0$$

where only W is involved. This observation is supported by recent numerical simulations of crack nucleation along interfaces with stochastic distributions of $\tau_{p}$ and homogeneous W (Albertini et al., 2020), except in rare situations where the asperity scale interacts with the nucleation length (Schär et al., 2021). One may then focus on variations of weakening rate W to quantify the influence of multiscale heterogeneities on fault stability.

### 2.2 Measuring the Nucleation Length in Presence of Weakening Rate Variations: A Model Fault Approach

**Figure 2:**
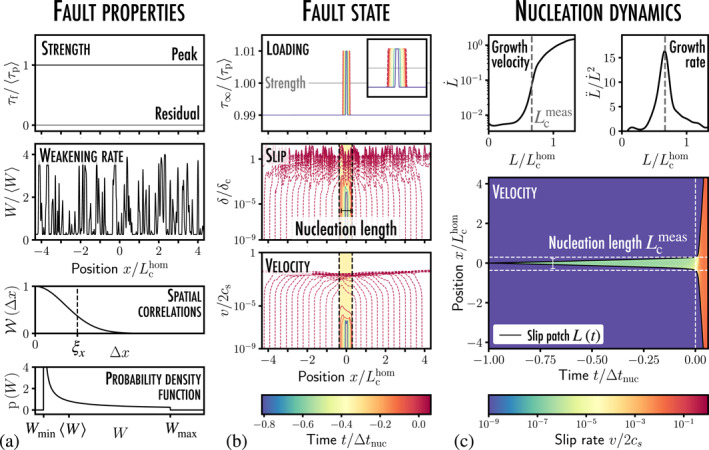
Measuring the nucleation length of a heterogeneous fault: (a) $\tau_{p}$ and $\tau_{r}$ are considered uniform along the interface, while the weakening rate W varies with the position. These variations occur over a characteristic length $\xi_{x}$ and are distributed following a beta distribution p(W) between two extremal values $\left[W_{\min},W_{\max}\right]$. (b) The model interface is loaded through an overstressed patch that slowly expands in time. A slip perturbation δ and an associated velocity perturbation $v=\dot{\delta }$ develop as time grows. The dynamics are characterized by two phases: the first phase consists of quasistatic growth (solid lines) and the second involves dynamic crack propagation (dashed lines) when the slipping region reaches a critical size $L_{c}$. (c) This shift in dynamics is observed on the temporal evolution of the slip patch size L(t) or its growth velocity $\dot{L}$. In particular, $\dot{L}$ hits an inflection point at $L=L_{c}$ (in linear‐log space), which provides an accurate measurement of $L_{c}$ from the growth rate $\ddot{L}/{\dot{L}}^{2}$. See Movie S2, Movie S3, and Figure S2 in the Supporting Information S1 for comparison with the homogeneous case of Uenishi and Rice (2003).

We thus focus on the stability behavior of an idealized fault along which both the peak $\tau_{p}$ and the residual friction $\tau_{r}$ are uniform (Figure 2a). To make any parallel to Mode I fracture easier and without any loss of generality, we set $\tau_{r}(x)=0$ (Albertini et al., 2020). Meanwhile, the weakening rate W may vary from several orders of magnitude along the fault. Following the procedure of Albertini et al., 2020 (see Section 1.1 in Supporting Information S1), we generate W fields that follow Gaussian correlations up to a characteristic length scale $\xi_{x}$. Moreover, the values of W follow a beta distribution of average 〈W〉 and standard deviation $\sigma_{w}$ between two extremal values $\left[W_{\min},W_{\max}\right]$. We set the nucleation length of the reference *homogeneous* material with uniform 〈W〉 as the adimensionalizing length of the system $L_{c}^{\mathrm{hom}}≃1.158\mu^{*}/\langle W\rangle$ (Uenishi & Rice, 2003). In the following, we consider $\sigma_{w}=\langle W\rangle$, $W_{\min}=0.25\langle W\rangle$, $W_{\max}=4\langle W\rangle$, and $\xi_{x}=0.05L_{c}^{\mathrm{hom}}$. The behavior of such a heterogeneous interface remains out of scope of the current theories of rupture nucleation where W is homogeneous (Albertini et al., 2020; Ampuero et al., 2006; Favreau et al., 1999; Uenishi & Rice, 2003). We then wonder how *local* variations of W as well as their intensity impact the *overall* fault stability.

In presence of spatial variations of W, the nucleation length is expected to fluctuate along the fault. In order to investigate the local instability dynamics, we force nucleation at a given point, referred to as “fault center,” by considering a macroscopic loading consisting of a slowly expanding region of size $L_{\sigma }=c_{\sigma }t$ ($c_{\sigma }\ll c_{s}$), where the stress locally exceeds the frictional resistance $\tau_{\infty }\geq \tau_{p}$ (Figure 2b). We observe in Figure 2b that a typical nucleation event is very similar, yet much simpler, to that of the heterogeneous fault of Section 2.1. Its dynamics consists of two distinct regimes: (a) the first regime involves a *stable quasistatic slip growth* for t<0 where a portion L of the interface is slipping, while (b) the second involves an *unstable dynamic crack propagation* for t>0 where a rupture front propagates until the whole fault is moving. The shift from one regime to another occurs when the slipping region outgrowths a critical length $L_{c}$ independently of the nature of the loading shape as long as it is *peaked* (see Figure S4 in Supporting Information S1). Importantly, this instability results from the collective motion of multiple asperities ($L_{c}≃13\xi_{x}$ in Figure 2).

To quantify the influence of spatial variations of W on $L_{c}$, we first propose a *heuristic* framework to measure it from numerical calculations. Looking at the evolution of the slip patch size L over time in Figure 2c, we observe that its growth velocity $\dot{L}$ follows an S‐shaped curve and hits an inflexion point (in linear‐log space) when $L=L_{c}$, as previously observed in laboratory experiments of earthquake nucleation between two polycarbonates blocks (Latour et al., 2013). The nucleation length $L_{c}$ may then be estimated from the maximal growth rate $\ddot{L}/{\dot{L}}^{2}$, as the length where the patch expansion is at its strongest. The validity of our heuristic approach is assessed on homogeneous interfaces for which $L_{c}^{\mathrm{hom}}≃1.158\mu^{*}/\langle W\rangle$ is known a priori (Favreau et al., 1999; Uenishi & Rice, 2003). We use it to numerically estimate the critical length $L_{c}$ of heterogeneous interfaces with a ±5% precision, corresponding to the error observed for the homogeneous interface of known $L_{c}$ (see Section 2.1 in Supporting Information S1).

## 3 Results and Discussion

### 3.1 Theoretical Model for Nucleation Length Predictions

Here, we propose a way to determine the critical length $L_{c}$ *analytically* building on both the theory of static friction and the physics of depinning. For a velocity perturbation v centered in $x=x_{0}$ with a support of size L, Equation 2 becomes:

(3) $$\frac{\mu^{*}}{2c_{s}}\frac{\partial v}{\partial t}(X,t)+\frac{2\mu^{*}}{L}L_{1}[v](X,t)-W(x_{0}+LX/2)v(X,t)=0$$

where $L_{1}[v](x,t)=\frac{1}{2\pi }\int_{-1}^{+1}\frac{\partial v/\partial X^{\prime }(X^{\prime },t)}{\left(X-X^{\prime }\right)}dX^{\prime }$ is the linear operator introduced in Dascalu et al. (2000) and Uenishi and Rice (2003), and $X=2(x-x_{0})/L$ is the reduced position.

To assess the fault stability, we perform a Linear Stability Analysis on Equation 3. It consists of finding the perturbation size L for which the linear symmetric operator $D\left[v\right](X)=\frac{2\mu^{*}}{L}L_{1}[v](X)-W(LX/2)v(X)$ admits a zero eigenvalue. Condensed matter physics provides one way to tackle this problem in the presence of heterogeneities (Cao et al., 2018; Démery et al., 2014; Tanguy & Vettorel, 2004): We expand the perturbation v with the disorder intensity $\sigma_{w}$ up to the second order $v=v_{0}+\sigma_{w}v_{1}+\sigma_{w}^{2}v_{2}$ and solve the eigenproblem D[v]=ωv, where $\omega =\omega_{0}+\sigma_{w}\omega_{1}+\sigma_{w}^{2}\omega_{2}$ (see Section 3 in Supporting Information S1). The nucleation length $L_{c}$ is the solution of the transcendental Equation 4, which encompasses the main novelty of the paper.

(4) $$\begin{array}{cccc} \frac{2\lambda_{0}\mu^{*}}{L_{c}(x_{0})} & - & \int_{-1}^{+1}W(x_{0}+L_{c}(x_{0})X^{\prime }/2)\nu_{0}{\left(X^{\prime }\right)}^{2}dX^{\prime } & \\ & - & \frac{L_{c}(x_{0})}{2\mu^{*}}\underset{1\leq k\leq k_{c}}{\sum }\frac{1}{\lambda_{k}-\lambda_{0}}{\left[\int_{-1}^{+1}W(x_{0}+L_{c}(x_{0})X^{\prime }/2)\nu_{0}(X^{\prime })\nu_{k}(X^{\prime })dX^{\prime }\right]}^{2}=0 & \end{array}$$

In Equation 4, $\nu_{k}$ denotes the $k^{\text{th}}$ eigenmode associated to the eigenvalue $\lambda_{k}$ of the homogeneous eigenproblem $L_{1}[v]=\lambda_{k}v$ (Dascalu et al., 2000; Uenishi & Rice, 2003). The first two terms of Equation 4 represent the heterogeneity contributions up to the first order. The value of the critical length $L_{c}$ at a position $x=x_{0}$ involves spatial variations of W on a scale potentially larger than the heterogeneity size $\xi_{x}$. This collective yet heterogeneous behavior in the earthquake nucleation cannot be grasped by the homogeneous W theory (Albertini et al., 2020; Uenishi & Rice, 2003). The third term corresponds to the second‐order contributions up to a critical mode $k_{c}≃2L_{c}/\xi_{x}$. This higher order term accounts for the influence of the spatial shape of W in all its complexity, beyond the special cases of periodic ordered distributions of asperities (Dublanchet, 2018; Perfettini et al., 2003; Ray & Viesca, 2017, 2019). Equation 4 is only valid as long as no point of the fault reaches its residual friction value (i.e., $\delta \left(x,t\right)<\delta_{c}\left(x\right)$). But W(x can be replaced by the instantaneous weakening rate W[x,δ(x,t)] to assess fault stability around a stable slip state δ(x,t) in the case of *nonlinear* or *piecewise linear* slip‐dependent friction. Yet, if Equation 4 gives *qualitative* information on the influence of a nonstationary weakening onto the nucleation process, it does not provide a *quantitative* framework to predict rupture nucleation for these more complex friction laws, as the slip evolution remains unknown.

**Figure 3:**
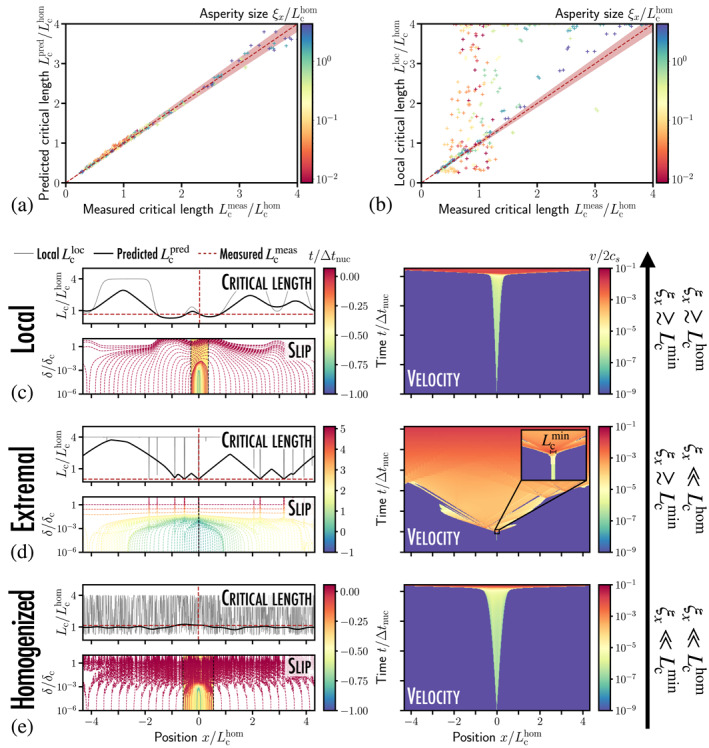
(a) The length of the critical instability nucleus $L_{c}^{\text{meas}}$ measured from the dynamic simulations is compared to the theoretical prediction $L_{c}^{\text{pred}}$ of Equation 4 at the fault center x=0 for a broad range of characteristic scale $\xi_{x}$ of the asperities (320 simulations). Red region: ±5% error on $L_{c}^{\text{meas}}$. (b) $L_{c}^{\text{meas}}$ may strongly differ from the local nucleation length of Uenishi and Rice (2003) $L_{c}^{\text{loc}}\left(x\right)≃1.158\mu^{*}/W\left(x\right)$ at x=0. The interplay between the length scales set by the frictional properties and the asperity size gives birth to three instability regimes: (c) When $\xi_{x}$ is larger than the homogeneous nucleation length $L_{c}^{\mathrm{hom}}≃1.158\mu^{*}/\langle W\rangle$ set by the average frictional properties, the effective nucleation length $L_{c}(x)$ of Equation 4 follows Uenishi and Rice (2003)'s predictions $L_{c}(0)≃L_{c}^{\text{loc}}(0)$; (d) when $\xi_{x}$ is smaller than $L_{c}^{\mathrm{hom}}$ yet larger than the minimal nucleation length $L_{c}^{\min}≃1.158\mu^{*}/W_{\max}$ set by the most brittle defect, $L_{c}(x)$ departs significantly from $L_{c}^{\text{loc}}(x)$ but can be locally controlled by the *extrema* of the weakening rate distribution $L_{c}(0)≃L_{c}^{\min}$. Inset: instability birth at $L≃L_{c}^{\min}=0.02L_{c}^{\mathrm{hom}}$; (e) when $\xi_{x}$ is smaller than both $L_{c}^{\mathrm{hom}}$ and $L_{c}^{\min}$, the nucleation behavior is *homogenized* and the nucleation length $L_{c}(x)$ is comparable to that set by the average frictional properties $L_{c}(x)≃L_{c}^{\mathrm{hom}}$ (see Movies [Link], [Link], [Link]).

When we compare the theoretical predictions $L_{c}^{\text{pred}}$ of Equation 4 to the numerically estimated critical lengths $L_{c}^{\text{meas}}$ for an asperity size $\xi_{x}$ that varies over four orders of magnitude, we observe an excellent agreement (Figure 3a). Note that (a) the nucleation length cannot be estimated from Uenishi and Rice (2003)'s homogeneous theory (Figure 3b) and (b) the second‐order contributions are required for accurate predictions (see Figure S7 in Supporting Information S1).

Equation 4 unveils rich physics about the impact of microscopic heterogeneities on the macroscopic fault stability. From it, one can directly link the spatial profiles of weakening rate W to the local evolution of the nucleation length $L_{c}$ along the interface (see black solid lines in Figures 3c–3e). In our simulations, the effective nucleation length corresponds to the one predicted at x=0 due to the *peaked* nature of the loading. In more realistic cases, the position $x_{0}$ of the earthquake nucleus (and the associated nucleation length $L_{c}(x_{0})$) will depend not only (a) on heterogeneities of peak strength $\tau_{p}$, but also (b) on the spatial shape of the macroscopic loading $\tau_{\infty }$, similarly to what has been observed for nonlinear slip‐weakening laws (Rice & Uenishi, 2010).

**Figure 4:**
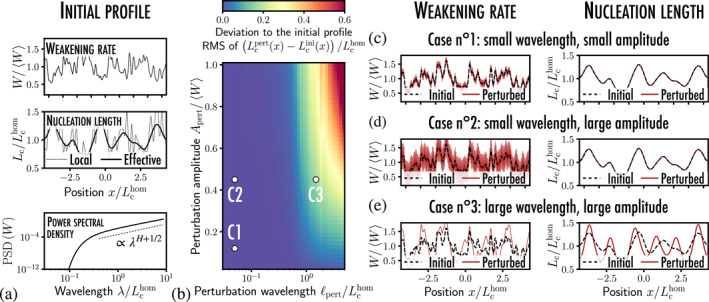
Influence of a modal perturbation on the stability of a heterogeneous fault: (a) an initial weakening rate profile $W_{\text{ini}}(x)$ consisting of the superposition of multiple spatial modes is considered. The spatial variations of $W_{\text{ini}}$ give birth to an effective profile of nucleation length $L_{c}^{\text{ini}}(x)$ following Equation 4. (b) The influence of the superposition of a unimodal perturbation $\hat{w}$ of period $\ell_{\text{pert}}$ and amplitude $A_{\text{pert}}$ to the initial W‐profile is quantified through the root‐mean‐square of the difference between the spatial profile of initial nucleation length $L_{c}^{\text{ini}}(x)$ related to $W_{\text{ini}}(x)$ and the perturbed one $L_{c}^{\text{pert}}(x)$ related to $W_{\text{ini}}(x)+\hat{w}(x)$. (c–e) When the perturbation wavelength $\ell_{\text{pert}}$ is smaller than the initial nucleation length $L_{c}^{\text{ini}}$, it does not change its spatial profile, no matter the perturbation amplitude. Only the larger wavelength perturbations may influence the nucleation length.

Overall, our framework provides ways to quantify the influence of a single heterogeneity on the fault stability depending on its size and intensity (see Figure 3) as well as that of the superposition of multiple perturbations of frictional properties (see Figure 4). Next, we build on Equation 4 and distinguish three instability regimes that can be linked to realistic earthquake nucleation scenarios on natural faults.

### 3.2 Instability Regimes in Earthquake Nucleation

In natural fault zones, heterogeneities in friction occur over many different scales. We observe them at the *centimetric* scale with minerals, clasts, and foliation, at the *metric/decametric* scale along large faults consisting of different lithologies, up to the scale of tectonic plates where *kilometric* asperities generated by a heterogeneous stress distribution have been suggested as potential nucleation sites for megathrust earthquakes in subduction zones. It is still uncertain how those different scales may interact with each other and how they ultimately impact the nucleation of earthquakes. Building on Equation 4, we highlight in Figures 3c–3e three different instability regimes, referred to as local, extremal, and homogenized regimes, respectively. They emerge from the interplay between three length scales: the heterogeneity size $\xi_{x}$, the nucleation length associated to average frictional properties $L_{c}^{\mathrm{hom}}≃1.158\mu^{*}/\langle W\rangle$, and the scale set by the weakest defect along the fault $L_{c}^{\min}≃1.158\mu^{*}/W_{\max}$.

1. Local regime: when $\xi_{x}≳L_{c}^{\mathrm{hom}}$ and $\xi ≳L_{c}^{\min}$, the weakening rate W is almost constant over the nucleation patch (see Figure 3c). One then retrieves the dynamics of homogeneous nucleation (Uenishi & Rice, 2003), and the effective nucleation length is set by the *local* frictional properties at the fault center $L_{c}(0)≃L_{c}^{\text{loc}}≃1.158\mu^{*}/W(0)$, which can be distributed above (W(0)<〈W〉) or below (W(0)>〈W〉) $L_{c}^{\mathrm{hom}}$
2. Extremal regime: when $\xi_{x}\ll L_{c}^{\mathrm{hom}}$ and $\xi_{x}≳L_{c}^{\min}$, a critical nucleation patch of size $L_{c}(0)≃L_{c}^{\min}$ may develop within a single brittle asperity of size $\xi_{x}$, where the weakening rate reaches its *maximal* value $W_{\max}$. This small event destabilizes the interface as a whole, generating a complex dynamics of multiple slip pulses (see velocity map in Figure 3d for which $\xi_{x}=0.01L_{c}^{\mathrm{hom}}=2L_{c}^{\min}$). Along natural faults, these small ruptures may be arrested by local barriers of strength $\tau_{p}$, but they may trigger a *cascade* of nucleation events centered on other weakest spots until the entire fault fails (de Geus et al., 2019; Noda et al., 2013; Zhang et al., 2003). Note that (a) these brittle asperities influence the effective nucleation length $L_{c}$ far away from them, and that, in contrast, and (b) other weak spots may not be critical if not brittle enough that is., $\xi_{x}\ll L_{c}^{\text{loc}}≳L_{c}^{\mathrm{hom}}$ (see the spatial evolution of $L_{c}^{\text{pred}}$ in black line in Figure 3d and Section 4 in Supporting Information S1)
3. Homogenized regime: when $\xi_{x}\ll L_{c}^{\mathrm{hom}}$ and $\xi_{x}\ll L_{c}^{\min}$, no critical slip patch can develop within a single asperity. Nucleation occurs after the collective depinning of multiple asperities (Dublanchet, 2018; Perfettini et al., 2003; Ray & Viesca, 2019) with dynamics similar to that of homogeneous nucleation. The critical length $L_{c}$ fluctuates around its *homogenized* value $L_{c}^{\mathrm{hom}}$ set by the averaged frictional properties (see Figure 3e) and can then be studied within the homogeneous nucleation theory of Uenishi and Rice (2003). Note that Equation 4 fully captures the fluctuations of $L_{c}$ around $L_{c}^{\mathrm{hom}}$, which may grow as the intensity $\sigma_{w}$ of weakening rate fluctuations increases

We argue here that all three instability regimes could occur along natural faults depending on their size, geometry, maturity, and lithology. But the *homogenized* regime proves to be of major importance for geophysical applications. Indeed, heterogeneous fracture is often described as a *critical* phenomenon controlled by the *weakest* defect, thus ruling out its study within a homogeneous framework. Yet, our results suggest that under the scale‐separation condition $\xi_{x}\ll L_{c}^{\min}$, the stability behavior of a heterogeneous fault can be studied within the homogeneous framework of Favreau et al. (1999) and Uenishi and Rice (2003) with $L_{c}≃L_{c}^{\mathrm{hom}}≃1.158\mu^{*}/\langle W\rangle$. Moreover, the existence of the *homogenized* regime may account for the relative reproductivity of laboratory experiments where sample roughness is often imposed and kept relatively smooth and further justifies their relevance in the modeling of natural faults.

### 3.3 Influence of Each Asperity Scale to the Global Stability of Heterogeneous Fault

So far, we considered cases where the distribution of weakening rate asperities could be described through a unique length scale $\xi_{x}$. Yet, the heterogeneities of weakening rate may emerge from, for example., the fault roughness that exhibits a scale‐free self‐affine behavior that spans over several decades of length scales (Candela et al., 2012), which makes the modeling of rough faults particularly challenging from a numerical point of view. Up to now, it is still largely unclear which length scales actively participate in the fault stability and which may be averaged in a realistic modeling of earthquake nucleation along rough faults.

To further demonstrate the potential of our theoretical framework, we consider a heterogeneous fault with a weakening rate profile $W_{\text{ini}}(x)$ (see Figure 4a) that emerges from a multiscale distribution of asperities with a Hurst exponent H=0.7 (Ampuero et al., 2006). The nucleation length $L_{c}^{\text{ini}}(x)$ can be computed from Equation 4. We superpose to the initial weakening rate profile $W_{\text{ini}}$, a unimodal perturbation $\hat{w}$ of period $\ell_{\text{pert}}$, and amplitude $A_{\text{pert}}$, giving birth to a perturbed profile $W_{\text{pert}}$:

(5) $$W_{\text{pert}}(x)=W_{\text{ini}}(x)+A_{\text{pert}}\cdot \cos(\frac{2\pi }{\ell_{\text{pert}}}x)$$

When computing the nucleation length $L_{c}^{\text{pert}}(x)$ associated to $W_{\text{pert}}(x)$, we observe that only perturbations whose wavelength is larger than the reference nucleation length $L_{c}^{\text{ini}}(x)$ matter (see Figure 4b and 4e), and that the perturbation of critical length increases with both the wavelength $\ell_{\text{pert}}$ and the amplitude $A_{\text{pert}}$. Furthermore, the small‐scale perturbations ($\ell_{\text{pert}}≲L_{c}^{\mathrm{hom}}$) do not change the nucleation length $L_{c}^{\text{pert}}$, whether its amplitude is small (Figure 4c) or large (Figure 4d). Note that if the initial nucleation length $L_{c}^{\text{ini}}$ locally drops to extremal values (see Figure 3d), very small‐scale asperities may then influence the overall stability behavior of the fault.

Overall, our work provides then *quantitative reasoning* to assess which scale of asperities should be included in the modeling of complex faults and which can be *averaged*, when the frictional heterogeneities span over several length scales.

## 4 Conclusion

Nucleation processes along fault with differential frictional weakening W are a *collective* phenomenon that may involve the progressive depinning of multiple asperities until a perturbation of size $L_{c}$ is reached. Building on the theory of static friction and the physics of depinning, we proposed an analytical framework that allows to predict the effective critical length $L_{c}$ for *any* spatial profile of W. This framework has been successfully compared to dynamic simulations of Mode II friction and is directly tractable to nucleation of Mode I and Mode III fracture along weak interfaces. It provides clues to explain various nucleation scenarios observed in laboratory experiments and in nature as well as to derive scale‐separation conditions assessing the influence of one asperity scale on the overall fault stability. Furthermore, it may provide ways to estimate the *shear loading levels* and the *position* at which nucleation occurs along more complex interfaces *of known frictional properties*, where all frictional quantities $\left(\tau_{p},\tau_{r},W\right)$ as well as the external loading $\left(\sigma_{n},\tau_{\infty }\right)$ fluctuate due to, for example, fault roughness (Cattania & Segall, 2021). The recent analogy drawn between earthquake nucleation for rate‐and‐state friction and rupture initiation along heterogeneous piecewise linear slip‐weakening interfaces (Ray & Viesca, 2017; Viesca, 2016a) provides convincing ways to extend the proposed framework to rate‐and‐state friction laws. The generalization of our results to (a) nonlinear slip‐dependent friction laws and (b) a three‐dimensional setting is not straightforward, but one may adapt the approach proposed in this work to handle fault inhomogeneities to the energetic nucleation framework of Rice and Uenishi (2010). Yet, further experimental work is needed to assess the validity of our framework in predicting the influence of heterogeneities on the nucleation process. Dynamic rupture experiments performed on model microarchitectured faults in the laboratory (e.g., Berman et al., 2020) may constitute a critical test for our theory.

## Supporting information

Supporting Information S1

Movie S1

Movie S2

Movie S3

Movie S4

Movie S5

Movie S6

## Data Availability

Data regarding the figures of the main text are available on Zenodo (Lebihain et al., 2021).
