# Supplementary material for: Earthquake Nucleation Along Faults With Heterogeneous Weakening Rate
Source: Geophys Res Lett. 2021 Nov 9;48(21):e2021GL094901. doi: 10.1029/2021GL094901 (PMC9286591; doi:10.1029/2021GL094901)
Supplement: Supplementary file 1 — Supporting Information S1 [file GRL-48-0-s007.pdf]

Supporting Information for "Earthquake Nucleation along Faults with Heterogeneous Weakening Rate"

Mathias Lebihain[1,3], Thibault Roch[2], Marie Violay[1], Jean-François Molinari[2]

[1]Laboratory of Experimental Rock Mechanics, Civil Engineering Institute, École Polytechnique Fédérale de Lausanne, Station 18, CH-1015 Lausanne, Switzerland

[2]Computational Solid Mechanics Laboratory, Civil Engineering Institute, Materials Science and Engineering Institute, École Polytechnique Fédérale de Lausanne, Station 18, CH-1015 Lausanne, Switzerland

[3]Laboratoire Navier, École des Ponts ParisTech, Université Gustave Eiffel, CNRS (UMR 8205), 6-8 avenue Blaise Pascal, 77455 Marne-la-Vallée, France

Contents of this file

1. Text S1 to S4
2. Figures S1 to S6

Additional Supporting Information (Files uploaded separately)

1. Movie S1 (*ms01.mp4*) for an example of nucleation instability along the heterogeneous fault of Fig. 1.

2. Movie S2 (*ms02.mp4*) for an example of nucleation instability along the heterogeneous model fault of Fig. 2.

3. Movie S3 (*ms03.mp4*) for an example of nucleation instability along a homogeneous reference fault.

4. Movie S4 (*ms04.mp4*) for an example of the local regime of Fig. 3.c.

5. Movie S5 (*ms05.mp4*) for an example of the extremal regime of Fig. 3.d.

6. Movie S6 (*ms06.mp4*) for an example of the homogenized regime of Fig. 3.e.

Introduction

We recall in S1 the basics of the numerical model used in this study. We describe in S2 how one may measure the nucleation length L_c from the dynamics of the main slip patch. We derive in S3 equation (4) that predicts the nucleation length of heterogeneous fault with a spatially varying weakening rate. In S4, we illustrate in greater details the spatial evolution of the nucleation length along the fault associated with the extremal regime of Section 3.2.

1. Numerical model

1.1. Generation of heterogeneous W profile

We build here on the method of (Albertini et al., 2020) to produce random fields of weakening rate $W(x)$ with controlled statistical properties. In the following, $\mathrm{E}[\cdot]$ denotes the expectation of a random variable over multiple disorder realizations. Moreover, we consider (i) *statistically homogeneous* and (ii) *ergodic* materials, so that ensemble averages $\mathrm{E}[\cdot]$ (i) do not depend on the observation point x, and (ii) are equivalent to spatial average $\langle\cdot\rangle$ over large enough interfaces.

Building on previous results of condensed matter physics (Tanguy & Vettorel, 2004; Démery et al., 2014), we expect that the stability behavior of the interface is likely

to be influenced by five statistical parameters of the random field W: (i) its average $\langle W \rangle = E[W]$, (ii) its standard deviation $\sigma_{\mathrm{w}} = \sqrt{E[W^2] - \langle W \rangle^2}$, (iii) its extremal values $W_{\min}$ and $W_{\max}$, (iv) its correlation length ξ_x and (v) its correlator shape $\mathcal{W}$ defined as $E[(W(x+\Delta x) - \langle W \rangle) \times (W(x) - \langle W \rangle)] = \sigma_{\mathrm{w}}^2 \mathcal{W}(|\Delta x| / \xi_x)$.

In order to generate a distribution of W values that is bounded between two extremal values $W_{\min}$ and $W_{\max}$, we write $W = W_{\min} + (W_{\max} - W_{\min}) w_\beta$, where w_β is a stochastic field following a beta distribution set by the two parameters (α, β). Then one gets:

$$\begin{cases} \alpha = \left(\frac{W_{\max} - W_{\min}}{\sigma_{\mathrm{w}}}\right)^2 \frac{\rho}{(1+\rho)^3} - \frac{1}{1+\rho} \\ \beta = \rho\alpha \end{cases} \quad \text{where } \rho = \frac{W_{\max} - \langle W \rangle}{\langle W \rangle - W_{\min}} \tag{1}$$

Note that Eq. S(1) requires $\alpha, \beta > 0$, a condition that constrains the value of the disorder parameters $(\langle W \rangle, \sigma_{\mathrm{w}}, W_{\min}, W_{\max})$.

The spatial random field w_β is generated following the procedure of (Albertini et al., 2020). First, a Gaussian random field w_g of zero average and unit variance is constructed using spectral representation (Shinozuka & Deodatis, 1991):

$$w_g(x) = \sum_{j=1}^{J} \sigma_j \left(A_j \cos(k_j x) + B_j \sin(k_j x)\right) \tag{2}$$

where A_j and B_j are independent Gaussian random variables with zero average and unit variance, $k_j = \frac{2\pi j}{L_x}$ is the modal wave-number, which is selected to ensure a L_x-periodicity, and $J = L_x/\Delta x$ where Δx is the step size along the interface. The modal variance σ_j^2 is computed from the disorder shape $\mathcal{W}$:

$$\sigma_j^2 = \tilde{\mathcal{W}}(k_j) / \sum_{j'=1}^{J} \tilde{\mathcal{W}}(k_{j'}) \tag{3}$$

where $\tilde{\mathcal{W}}(k)$ is the Fourier transform of the disorder shape $\mathcal{W}$, $\tilde{\mathcal{W}}(k) = \int_{-\infty}^{+\infty} \mathcal{W}(x)\, e^{-ikx} dx$. Here, we choose Gaussian correlations $\mathcal{W}(\Delta x/\xi_x) = e^{-(\Delta x/\xi_x)^2}$, i.e. $\tilde{\mathcal{W}}(k) = \xi_x e^{-(\xi_x k)^2/4}$. Note that, as we will see in the following, this correlation shape is expected to influence the nucleation behavior (see Eq. S(29)).

We finally apply a non-linear mapping to w_g from the Gaussian cumulative distribution function to the one of the beta distribution to get w_σ. This transformation is expected not to affect much $\mathcal{W}$ and ξ_x as long as $\mathcal{W}$ is positive (Grigoriu, 2002). Examples of W fields are given in Fig. S1 for three different correlation lengths ξ_x.

1.2. Dynamic simulations of Mode II fracture along cohesive interfaces

The numerical method used in this work is based on a spectral boundary integral formulation of the elastodynamic equations relating the tractions τ acting at the interface between two semi-infinite elastic solids, and the resulting slip δ at the interface, as presented by (Geubelle & Rice, 1995) and (Breitenfeld & Geubelle, 1998). The numerical computations conducted in this work use an open-source implementation of the spectral boundary integral formulation. Here, we summarize the method in the case of a pure in-plane shear solicitation (Mode II) in 2D.

$$\tau(x,t) = \tau_\infty(x,t) - \frac{\mu^*}{2c_\mathrm{s}} \frac{\partial \delta}{\partial t}(x,t) + s(x,t) \tag{4}$$

The first right-hand side τ_∞ corresponds to the macroscopic far-field loading, and physically represents the stresses that would exist in the absence of a crack. The second one $-\frac{\mu^*}{2c_\mathrm{s}}\frac{\partial \delta}{\partial t}$ corresponds to the instantaneous response to a change in sliding velocity, often referred to as "*radiation damping*", and physically represents the energy radiated from

the interface to the bulk through elastic waves. The last term s corresponds to a wave-mediated stress transfer that relates to the full history of the slip δ along the interface. When the interface has been initially at rest for a time much larger than the characteristic one $t^* \simeq L_x/c_\mathrm{s}$ set by the wave motion, the term s reduces to the operator $-\mu^*\mathcal{L}[\delta]$ that characterizes static stress redistribution. One then gets the quasi-dynamic left hand side of Eq. (1) from the fully dynamic Eq. S(4). Note that the term $s(x,t)$ is related in the spectral domain to the slip history $\delta(x,t)$ via a convolution integral whose expression can be found in (Breitenfeld & Geubelle, 1998).

The continuity of displacements and velocities at the interface is satisfied as long as the stresses are lower than the interface strength τ_p. Otherwise, fracture occurs, and the interface velocity $v = \dot{\delta}$ is found such that the stresses computed with Eq. S(4) and the frictional stresses τ_f are in equilibrium. In this work, we consider the simple case of a linear slip-dependent friction:

$$\tau_\mathrm{f}(x,t) = \max\left[\tau_\mathrm{p}(x) - W(x)\,\delta(x,t)\,,\tau_\mathrm{r}(x)\right] \tag{5}$$

where τ_p is the peak friction, τ_r the residual one, and $W = -\partial\tau_\mathrm{f}/\partial\delta$ is the weakening rate. Note that the sign of W is not prescribed in the proposed theoretical framework, but, our simulations only examine cases where $W(x) \geq 0$.

After solving the interface conditions, the elastodynamic relations are integrated in time using an explicit time-stepping as:

$$\delta(x,t+\Delta t) = \delta(x,t) + v(x,t)\,\Delta t \tag{6}$$

with the time step $\Delta t = \beta \Delta x / c_\mathrm{s}$ being a fraction of the time required for a shear wave to travel through one unit of the spatial discretization spacing Δx. In our simulations, we set $\Delta x = \min\left(\xi_x/8, L_\mathrm{c}^\mathrm{min}/20\right)$, in order to have both a proper representation of the material disorder ($\simeq$ 8 points by heterogeneity) and a correct description of the extremal nucleation events (Lapusta et al., 2000) ($\simeq$ 20 points in the smallest nucleation patch of size $L_\mathrm{c}^\mathrm{min} \simeq 1.158\mu^*/W_\mathrm{max} = 0.01 L_\mathrm{c}^\mathrm{hom}$ reached in the extremal case of Section 3.2). The parameter β is set to $\beta = 0.3$ so that the stability of the scheme is guaranteed.

2. Identifying the critical length in numerical calculations

2.1. Measurement of L_c from slip patch dynamics

The crucial variable discussed in our work is the critical length L_c that marks the onset of the instability. Being able to properly measure L_c is still a challenge both numerically and experimentally. A previous study proposed to determine this critical length in the rate-and-state framework investigating the stability of initial velocity perturbations of fixed length (Brener et al., 2018). Here, we get closer to experimental conditions, and consider nucleation from a slowly increasing macroscopic shear loading. We force nucleation at a given location, referred to as "fault center" in the following, by loading the fault with a slowly expanding overstressed patch. We identify L_c as the size L of the slipping region when it shifts from quasi-static stable slip growth to dynamic unstable crack propagation (see Fig. S3). Such an evolution can be accessed experimentally either looking at the evolution of the contact area (Ben-David & Fineberg, 2011; Svetlizky et al., 2016) or from photo-elasticity (Latour et al., 2013).

We observe in Fig. 2, S2 and S3 that the slip patch evolution $L(t)$ allows for a precise determination of the critical time to instability t_c, but that the accurate determination of L_c proves trickier due to the smooth transition from the quasi-static regime to the dynamic one (Ben-David & Fineberg, 2011). Yet, if one looks at its growth velocity $\dot{L}$, it follows a characteristic S-shaped curve that has been previously observed in experiments of earthquake nucleation between two polycarbonates blocks (Latour et al., 2013). Building on simulations on a homogeneous fault $W(x) = \langle W \rangle$ for which the critical length is known $L_c = 1.158\mu^*/\langle W \rangle$, we see that the critical length L_c is reached when $\dot{L}$ hits an inflection point (in linear-log) (see Fig. S2.d), while experimental observations where L_c was not known *a priori* suggested that L_c may correspond to the low end of the S-shape (Latour et al., 2013). It provides an accurate measurement of the critical length from the maximal growth rate $\ddot{L}/\dot{L}^2$ (Fig. S2.e) even in presence of heterogeneities, since the evolution of the slipping region remains similar although noisier due to local asperities (Fig. S3).

Note that we measure the signal $L(t)$ from the instantaneous interface state (where $\delta > 0$) in our simulations. This signal proves not smooth but rather *noisy*, since it is composed of jumps of size Δx, the spatial grid size. Thus, the evaluation of the growth velocity $\dot{L}$ and the growth rate $\ddot{L}/\dot{L}^2$ cannot be performed through standard finite differences (cross black points in Fig. S2.d and Fig. S3.d). We rather compute $\dot{L}$ and $\ddot{L}/\dot{L}^2$ through a *local weighted least-square procedure* on a moving portion ΔL of the $L(t)$ signal (dashed red lines in Fig. S2.d and Fig. S3.d). This method is traditionally used in analyzing experimental data to access partial derivatives of noisy signals (Breitkopf et al., 2005). For each simulation, the maximum of the growth rate $\ddot{L}/\dot{L}^2$ is pinpointed manually

for three regularizing length $\Delta L = 0.4/0.5/0.6 L_{\rm c}^{\rm hom}$, providing error bars in the numerical measurement of $L_{\rm c}$. For all 320 considered simulations, our procedure allows to estimate $L_{\rm c}$ with a $\pm 5\%$ precision, which corresponds to the error observed on a homogeneous fault for which the nucleation length is given analytically by (Uenishi & Rice, 2003)'s theory (Fig. S2).

2.2. Independence of $L_{\rm c}$ with the loading shape

Note that the measured critical length $L_{\rm c}$ does not depend on the loading shape (crenel-like in Fig. S4.a or linearly peaked in Fig. S4.b) as long as it is *peaked* on a given fault location. The proposed identification procedure may provide a robust method to identify the critical length $L_{\rm c}$ in laboratory experiments of earthquake nucleation.

3. Linear stability analysis in presence of disorder

We present here the linear stability analysis that results in the determination of the critical length $L_{\rm c}$ of Eq. (4). Our approach mainly borrows ideas from (Uenishi & Rice, 2003)'s study of earthquake nucleation in static friction, and (Tanguy & Vettorel, 2004)'s paper that investigates the onset of instability along disordered elastic interfaces.

3.1. Master equation

Following theoretical works on earthquake nucleation along a frictional interface (Uenishi & Rice, 2003; Rubin & Ampuero, 2005; Viesca, 2016a, 2016b), we consider a velocity perturbation v centered in $x = 0$ of support L ($v = 0\,, \forall x \in (-\infty, -L/2] \cup [L/2, +\infty)$). As explained in the main text, the stress balance of Eq. (1) ultimately

reduces to:

$$\frac{\mu^*}{2c_{\rm s}}\frac{\partial v}{\partial t}(X,t)+\frac{2\mu^*}{L}\mathcal{L}_1[v](X,t)-W(LX/2)\,v(X,t)=0 \tag{7}$$

where $X=2x/L$ is the reduced position, and $\mathcal{L}_1[\delta](X,t)=\frac{1}{2\pi}\int_{-1}^{+1}\frac{\partial\delta/\partial X'(X',t)}{(X-X')}dX'$ is a non-local operator emerging from the static stress transfer for an infinite 1D planar fault $\mathcal{L}[\delta](X,t)=\frac{1}{2\pi}\int_{-\infty}^{+\infty}\frac{\partial\delta/\partial X'(X',t)}{(X-X')}dX'$. Note that the operator $\mathcal{L}$ usually writes as $\mathcal{L}[\delta](X,t)=\frac{1}{2\pi}\mathrm{PV}\int_{-\infty}^{+\infty}\frac{\delta(X,t)-\delta(X',t)}{(X-X')^2}dX'$ in the physics of disordered elastic media (Tanguy & Vettorel, 2004; Démery et al., 2014; Cao et al., 2018), where PV denotes a Cauchy principal value.

It is clear from Eq. S(7) that the stability of the interface is directly assessed by the positivity of the eigenvalues of the *linear symmetric operator* $\mathcal{D}$:

$$\mathcal{D}[v](X)=\frac{2\mu^*}{L}\mathcal{L}_1[v](X)-W(LX/2)\,v(X) \tag{8}$$

Our linear stability analysis then reduces to find the eigenvalues (ω_k) of the operator $\mathcal{D}$.

3.2. Auxiliary homogeneous eigenproblem

In order to find the eigenvalues of the disordered operator $\mathcal{D}$, it is of particular interest to study first the homogeneous operator $\mathcal{H}$:

$$\mathcal{H}[v](X)=\frac{2\mu^*}{L}\mathcal{L}_1[v](X)-\langle W\rangle\,v(X) \tag{9}$$

The eigenvalues (h_k) of the linear symmetric operator are directly linked to those (λ_k) of $\mathcal{L}_1$ through the equation:

$$h_k=\frac{2\mu^*}{L}\lambda_k-\langle W\rangle \tag{10}$$

We observe that a homogeneous fault is unconditionally stable for slip-strengthening and slip-neutral materials ($\langle W \rangle \geq 0$), and stable up to $L = L_{\mathrm{c}}^{\mathrm{hom}} = 2\lambda_0\mu^*/\langle W \rangle \simeq 1.158\mu^*/\langle W \rangle$ for slip-weakening materials ($\langle W \rangle < 0$).

The operator $\mathcal{L}_1$ has been thoroughly studied by (Dascalu et al., 2000) and (Uenishi & Rice, 2003). $\mathcal{L}_1$ is self-adjoint and positive definite, implying that all its eigenvalues (λ_k) are positive $0 < \lambda_0 < ... < \lambda_n < \lambda_{n+1} < ...$ and that the associated eigenfunctions (ν_k) are orthogonal:

$$(\nu_k, \nu_{k'}) = \int_{-1}^{+1} \nu_k(X)\, \nu_{k'}(X)\, dX = \Delta(k - k') \tag{11}$$

where Δ is the Dirac function. The eigenvalues (λ_k) and the spatial form of the eigenfunctions $(\nu_k(X))$ can be computed from (Dascalu et al., 2000)'s Appendix A. Note that numerical computations of the (λ_k) and (ν_k) shows that $\lambda_k \simeq \lambda_0 + k\frac{\pi}{4} \simeq 0.5789 + k\frac{\pi}{4}$, and that $\nu_k(X) \simeq \cos\left(\frac{\pi}{2}(k+1)X + k\frac{\pi}{2}\right)$ for $k \gg 1$.

3.3. Disordered eigenproblem

We now focus on the disordered eigenproblem related to $\mathcal{D}$. Due to $\mathcal{L}_1$ being positive definite (Uenishi & Rice, 2003), $\mathcal{D} - W_{\mathrm{max}}\mathrm{Id}$ is also a *positive definite operator*. It implies that all eigenvalues (ω_k) of $\mathcal{D}$ are distinct from one another, and that the associated eigenfunctions (v_k) are orthogonal.

The next step consists in finding the (ω_q) and associated (v_k) satisfying:

$$\mathcal{D}[v_k](X) = \omega_k v_k(X) \tag{12}$$

building on the set of eigenfunctions (ν_k) associated to the homogeneous eigenproblem.

First, we write $W(x) = \langle W \rangle + \sigma_{\mathrm{w}} w(x)$, where again $\langle W \rangle$ is the average of the W field, σ_{w} its standard deviation, and w is a stochastic field of zero average and unit value. Note that $E[w(x+\Delta x) w(x)] = \mathcal{W}(|\Delta x| / \xi_x)$. We now follow the works of (Tanguy & Vettorel, 2004) and (Démery et al., 2014), and expand the spatial shape of the eigenfunction v_k at second order with the material disorder σ_{w}, $v_k = v_k^0 + \sigma_{\mathrm{w}} v_k^1 + \sigma_{\mathrm{w}}^2 v_k^2$. Since the associated eigenvalue ω_k is defined from v_k, ω_k is also expanded at second order $\omega = \omega_k^0 + \sigma_{\mathrm{w}} \omega_k^1 + \sigma_{\mathrm{w}}^2 \omega_k^2$.

Zero order terms Eq. S(12) gives at zero order:

$$\frac{2\mu^*}{L} \mathcal{L}_1\left(v_k^0\right)(X) - \langle W \rangle v_k^0(X) = \omega_k^0 v_k^0(X) \tag{13}$$

Expanding v_k^0 on the (ν_k), $v_k^0 = \sum_{k'=0}^{+\infty} V_{k,k'}^0 \nu_{k'}$, and taking the scalar product with the k'th eigenfunction ν_k, one gets:

$$\left(\omega_k^0 - \frac{2\mu^*}{L} \lambda_k + \langle W \rangle\right) V_{k,k'}^0 = 0 \tag{14}$$

As expected from the homogeneous zero order, v_k^0 only expands along one eigenfunction ν_q. We can always change the value of k to take it equal to q at the expense of the order between the (ω_k), i.e. ω_0 does not necessarily correspond to the smallest eigenvalue of $\mathcal{D}$. Ultimately :

$$v_k^0(X) = V_{k,k}^0 \nu_k(X) \tag{15}$$

and:

$$\omega_k^0 = \lambda_k \frac{2\mu^*}{L} - \langle W \rangle \tag{16}$$

Note that we find back the solution of (Uenishi & Rice, 2003) for the homogeneous reference material.

First order terms

$$\omega_k^1 v_k^0 (X) + \omega_k^0 v_k^1 (X) = \frac{2\mu^*}{L} \mathcal{L}_1 \left(v_k^1 \right) (X) - \langle W \rangle v_k^1 (X) - w (LX/2) v_k^0 (X) \qquad (17)$$

Again, we expand v_1 on the eigenfunctions $v_k^1 = \sum_{k'=0}^{+\infty} V_{k,k'}^1 \nu_{k'}$. Using Eq. S(15) and S(16) , this equation reduces to:

$$\omega_k^1 V_{k,k}^0 \nu_k (X) = \frac{2\mu^*}{L} \sum_{k'} (\lambda_{k'} - \lambda_k) V_{k,k'}^1 \nu_{k'} (X) - w (LX/2) V_{k,k}^0 \nu_k (X) \qquad (18)$$

Taking first the scalar product with the kth eigenfunction ν_k, one gets the first order contribution to the kth eigenvalue:

$$\omega_k^1 = -w_{kk} (L) \qquad (19)$$

where $w_{kk'} (L) = \langle w\nu_k, \nu_{k'} \rangle = \int_{-1}^{+1} w (LX'/2) \nu_k (X') \nu_{k'} (X') dX'$

Taking the scalar product with the k'th eigenfunction $\nu_{k'}$, one gets the coefficient $V_{k,k'}^1$ for $k' \neq k$:

$$V_{k,k'}^1 = V_{k,k}^0 \frac{L}{2\mu^*} \frac{w_{kk'} (L)}{\lambda_{k'} - \lambda_k} \qquad (20)$$

and since $V_{k,k}^1$ is not prescribed, we take $V_{k,k}^1 = 0$ for simplicity.

Second order terms

$$\omega_k^2 v_k^0 (X) + \omega_k^1 v_k^1 (X) + \omega_k^0 v_k^2 (X) = \frac{2\mu^*}{L} \mathcal{L}_1 \left(v_k^2 \right) (X) - \langle W \rangle v_k^2 (X) - w (LX/2) v_k^1 (X) \qquad (21)$$

We expand v_k^2 on the eigenfunctions $v_k^2 = \sum_{k'=0}^{+\infty} V_{k,k'}^2 \nu_{k'}$. Using Eq. S(15) and S(16), it reduces to:

$$\omega_k^2 V_{k,k}^0 \nu_k (X) + \omega_k^1 \sum_{k'} V_{k,k'}^1 \nu_{k'} (X) = \frac{2\mu^*}{L} \sum_{k'} (\lambda_{k'} - \lambda_k) V_{k,k'}^2 \nu_{k'} (X) - w (LX/2) \sum_{k'} V_{k,k'}^1 \nu_{k'} (X) \qquad (22)$$

Taking the scalar product with the kth eigenfunction ν_k, and using Eq. S(19) and Eq. S(20), one gets the second order contribution to the kth eigenvalue:

$$\omega_k^2 = -\frac{L}{2\mu^*} \sum_{k' \neq k} \frac{w_{kk'}(L)^2}{\lambda_{k'} - \lambda_k} \tag{23}$$

Taking the scalar product with the k'th eigenfunction $\nu_{k'}$, one gets the coefficient $V^2_{k,k'}$ for $k' \neq k$:

$$V^2_{k,k'} = V^0_{k,k} \left(\frac{L}{2\mu^*}\right)^2 \frac{1}{\lambda_{k'} - \lambda_k} \left[\sum_{k'' \neq k,k'} \frac{w_{kk''}(L)\, w_{k'k''}(L)}{\lambda_{k''} - \lambda_k} + \frac{w_{k'k'}(L) - w_{kk}(L)}{\lambda_{k'} - \lambda_k} w_{kk'}(L) \right] \tag{24}$$

The kth eigenvalue ω_k finally reads:

$$\omega_k(L) = \frac{2\lambda_k \mu^*}{L} - \langle W \rangle - \sigma_{\text{w}} w_{kk}(L) - \sigma^2_{\text{w}} \frac{L}{2\mu^*} \sum_{k' \neq k} \frac{w_{kk'}(L)^2}{\lambda_{k'} - \lambda_k} \tag{25}$$

which writes also as:

$$\omega_k(L) = \frac{2\lambda_k \mu^*}{L} - \int_{-1}^{+1} W(LX'/2)\, \nu_k(X')^2\, dX' - \frac{L}{2\mu^*} \sum_{k' \neq k} \frac{1}{\lambda_{k'} - \lambda_k} \left[\int_{-1}^{+1} W(LX'/2)\, \nu_k(X')\, \nu_{k'}(X')\, dX' \right]^2 \tag{26}$$

Note that the first two terms of Eq. S(26) consist of the zero and first order contribution of the material disorder w to the interface stability, while the third term encompasses second-order contributions.

3.4. Numerical evaluation of Eq. (4)

The modal critical length L^k_{c} can then be found from Eq. S(26) as the first zero of $\omega_k(L)$. Yet, it involves an infinite sum of the disorder W contributions over all the eigenmodes (ν_k) of the homogeneous problem, which makes it impossible to compute numerically through standard root finding algorithms. We show here that one can only consider the

disorder contributions up to a critical mode $k_\mathrm{c} \simeq \lceil 2L/\xi_x \rceil$ to estimate the second order contributions with enough accuracy.

In order to do that, we need to assess how $w_{kk'}(L) = \int_{-1}^{+1} w(LX'/2)\,\nu_k(X')\,\nu_{k'}(X')\,dX'$ varies with $k' \neq k$. We now distinguish three cases:

• $\mathbf{\xi_x/L \gtrsim 1}$: in that case, $w(x)$ is almost constant over the interval $[-L/2, L/2]$. Thus, building on the orthogonality of the eigenfunctions (ν_k):

$$w_{kk'}(L) \simeq 0 \tag{27}$$

• $\mathbf{\xi_x/L \ll 1}$ and $\mathbf{\xi_x/L \ll 2/(k'+1)}$: here we can assume that there are enough heterogeneities of size ξ_x in the patch of size L to make spatial (and thus ensemble) averages relevant. Then:

$$\begin{aligned} w_{kk'}(L)^2 &\simeq E\left[w_{kk'}(L)^2\right] \\ &= \int_{-1}^{+1}\int_{-1}^{+1} E\left[w(LX/2)\,w(LX'/2)\right]\nu_k(X)\,\nu_{k'}(X)\,\nu_k(X')\,\nu_{k'}(X')\,dX\,dX' \\ &= \int_{-1}^{+1}\int_{-1}^{+1} \mathcal{W}(L|X-X'|/2\xi_x)\,\nu_k(X)\,\nu_{k'}(X)\,\nu_k(X')\,\nu_{k'}(X')\,dX\,dX' \\ &= \int_{-1}^{+1}\int_{s_r^{\min}}^{s_r^{\max}} \mathcal{W}(L|s|/\xi_x)\,\nu_k(r+s)\,\nu_{k'}(r+s)\,\nu_k(r-s)\,\nu_{k'}(r-s)\,ds\,dr \end{aligned} \tag{28}$$

where $s = (X-X')/2$ and $r = (X+X')/2$. $s_r^{\min}$ and $s_r^{\max}$ are the minimum and maximum values of s allowed by the inequalities $-1 \leq r-s \leq 1$ and $-1 \leq r+s \leq 1$. An example of such integration bounds is plotted in Fig. S5.a.

Following (Perrin & Rice, 1994), the interval over which $\mathcal{W}(L|s|/\xi_x)$ takes non-negligible values is "concentrated" near the center of the interval of integration $\left[s_r^{\min}, s_r^{\max}\right]$ along a zone of size ξ_x/L (see Fig. S5.a). Moreover, $\nu_k(r \pm s)$ and $\nu_{k'}(r \pm s)$, which are similar to

sinusoidal functions of half-period $2/(k+1)$ and $2/(k'+1)$, are almost constant where $\mathcal{W}(L|s|/\xi_x)$ is taking non-negligible values ($2/(k+1) \lesssim \xi_x/L$). Then, one may safely extend the last integral of Eq. S(28) from the interval $\left[s_r^{\min}, s_r^{\max}\right]$ to the entire real line; indeed, $s_r^{\min}$ and $s_r^{\max}$ are large for almost every r in the interval $[-1,1]$ when $L/\xi_x \ll 1$. It ultimately gives:

$$w_{kk'}(L)^2 \simeq \frac{\xi_x}{L}\tilde{\mathcal{W}}(0) \tag{29}$$

where $\tilde{\mathcal{W}}$ is the x-Fourier transform of the function $\mathcal{W}$.

- $\boldsymbol{\xi_x/L \ll 1}$ **and** $\boldsymbol{\xi_x/L \gg 2/(k'+1)}$: then $\nu_{k'}(r+\cdot)\,\nu_{k'}(r-\cdot)$ of Eq. S(28) is a sinusoidal function of half-period $L_{k'} = 4/(k'+1) \ll \xi_x/L$. Assuming, $k \ll k'$, $\mathcal{W}(L|s|/\xi_x)\,\nu_k(r+s)\,\nu_k(r-s)$ is almost constant on a half-period $L_{k'}$. It gives:

$$w_{kk'}(L)^2 \simeq 0 \tag{30}$$

One can then sum up to $k_c \simeq \lceil 2L/\xi_x \rceil$ and still grasp all the modal contributions of Eq. S(26). Note that this reasoning stands for $2L/\xi_x \gg 1$, and we recommend to take $k_c \geq 30$ for $2L/\xi_x \lesssim 1$. The validity of our reasoning is assessed in Fig. S5.b&c.

Using Eqs. S(23), S(27), S(29) & S(30), and the facts that $\lambda_k \simeq \lambda_0 + k\frac{\pi}{4}$ and $\sum_{k=1}^{n} \frac{1}{k} \underset{n \gg 1}{\sim} \ln(n)$, we find $\omega_k^2(L) \underset{L/\xi_x \gg 1}{\sim} -\frac{\sigma_w^2 \xi_x}{2\mu^*}\tilde{\mathcal{W}}(0)\ln(L/\xi_x)$. It provides a theoretical basis for the log-dependence of the second order eigenvalue ω_k^2 with the instability length observed in numerical simulations of disordered elastic systems (Tanguy & Vettorel, 2004). Note that (Tanguy & Vettorel, 2004) used a different approach (based on a Fourier analysis) to study the interface stability instead, while we build on the homogeneous eigenproblem of static friction (Dascalu et al., 2000; Uenishi & Rice, 2003).

3.5. Selected eigenmode

One last step is required to get our master Eq. (4) from Eq. S(26). Indeed, our last reasoning shows that $\omega_k(L) \underset{L/\xi_x \ll 1}{\sim} \frac{2\lambda_k \mu^*}{L} + W(0) > 0$, and $\omega_k(L) \underset{L/\xi_x \gg 1}{\sim} -\frac{\sigma_{\mathrm{w}}^2}{2\mu^*}\xi_x \ln(L/\xi_x) < 0$. Thus a modal instability length L_{c}^k that satisfies $\omega_k\left(L_{\mathrm{c}}^k\right) = 0$ is reached as the size L of the perturbation grows. Naturally, the critical length corresponds to the smallest modal instability length L_{c}^k. Numerical evaluations of Eq. S(26) for $k \in [0, 3]$ for 45'000 nucleation events makes us believe that the first mode $k = 0$ may always corresponds to the critical one (Fig. S6.d), as it is the case for homogeneous faults (Uenishi & Rice, 2003). It provides the last argument required for Eq. (4).

3.6. Comparison between 1st and 2nd order theories

If one chooses to overlook the influence of the collective depinning of the asperities during a nucleation event, and estimates the critical length L_{c} from its local value at the fault center $L_{\mathrm{c}}^{\mathrm{loc}} \simeq -2\lambda_0\mu^*/W(0)$, one does large errors on the nucleation dynamics (Fig. S7.a).

Thus, one needs to take into account the influence of the spatial variations of W. As it has been explained before, Eq. (4) is the sum of zero and first order contributions (two first terms) as well as second order ones (third term). The contributions up to the first order are much easier to compute since they only involve one eigenfunction ν_0, which is already used to predict the loading level and the position of nucleation event along heterogeneous interfaces with spatially varying strength/loading (Uenishi & Rice, 2003; Ampuero et al., 2006; Albertini et al., 2020). Thus, it might be seducing to measure the

critical length L_c from:

$$\frac{2\lambda_0\mu^*}{L_\mathrm{c}} - \int_{-1}^{+1} W\left(L_\mathrm{c}X'/2\right)\nu_0\left(X'\right)^2 dX' = 0 \tag{31}$$

We observe in Fig. S7.b that we underestimate L_c using Eq. S(31), and that the second order contributions of Eq. (4) are required for accurate precision.

4. Illustration of L_c variations for the extremal regime

The extremal regime Section 3.2 display very strong variations of nucleation length $L_\mathrm{c}^\mathrm{pred}(x)$ along the fault, which are predicted from Eq. (4). In particular, we see that some weak asperities, where $L_\mathrm{c}^\mathrm{loc}$ drops significantly due to the locally strong weakening rate (see Fig. S8.b), decrease the effective nucleation length $L_\mathrm{c}^\mathrm{pred}$ (e.g. the case of Fig. S8.b at $x = 0$), while other do not influence it significantly (e.g. the case of Fig. S8.d at $x = -1.82L_\mathrm{c}^\mathrm{hom}$). To illustrate the fault stability behavior, and further validate our semi-analytical framework, we will make rupture nucleate along different position along the "extremal" fault. The simulations have been performed under the following set of disorder parameters: $\sigma_\mathrm{w} = 8\,\langle W\rangle$, $W_\mathrm{min} = 0.25\,\langle W\rangle$, $W_\mathrm{max} = 100\,\langle W\rangle$, and $\xi_x = 0.02L_\mathrm{c}^\mathrm{hom}$.

First, we trigger in Fig. S8.c the instability at $x_0 = 0$, which corresponds to the extremal case of Fig. 3d. In this case, nucleation occurs on a single asperity, which is referred to as "*critical*" since an instability of critical size $L_\mathrm{c}^\mathrm{min} = 0.01L_\mathrm{c}^\mathrm{hom}$ can develop within the ξ_x-large defect ($\xi_x = 0.02L_\mathrm{c}^\mathrm{hom}$). This case is analogous to that of (Uenishi & Rice, 2003), and the measured nucleation length $L_\mathrm{c}^\mathrm{meas}$ corresponds to its local value $L_\mathrm{c}^\mathrm{loc}\left(x_0\right) = 1.158\mu^*/W\left(x_0\right) = L_\mathrm{c}^\mathrm{min}$.

Second, nucleation is forced at $x_0 \simeq 0.58L_c^{\mathrm{hom}}$, on the right of the critical asperity considered previously (see Fig. S8.d). In this case, the instability occurs when the slipping region reaches the critical asperity, so that the *effective* nucleation length L_c corresponds to the distance to the nearest neighboring extremely weak asperity. The measured nucleation length $L_c^{\mathrm{meas}} = 1.18Lc^{\mathrm{hom}}$ is accurately predicted by our model $L_c^{\mathrm{pred}}(x_0) = 1.23Lc^{\mathrm{hom}}$, even if it does not strictly encompass any notion of distance to critical asperities.

Third, we trigger the nucleation at $x_0 \simeq -1.82L_c^{\mathrm{hom}}$, where the local critical length – computed from (Uenishi & Rice, 2003)'s theory – drops to $L_c^{\mathrm{loc}}(x_0) \simeq 1.158\mu^*/W(x_0) \simeq 0.08L_c^{\mathrm{hom}}$. Despite the presence of a very weak asperity at the center of the slip patch, the instability occurs when the slip patch reaches a value $L_c^{\mathrm{meas}} = 1.96L_c^{\mathrm{hom}}$, much larger than $L_c^{\mathrm{loc}}(x_0)$. A prolonged stability was expected since the asperity is too small ($\xi_x = 0.02L_c^{\mathrm{hom}}$) for a instability to develop within it ($L_c^{\mathrm{loc}}(x_0) \simeq 0.08L_c^{\mathrm{hom}}$). This feature is grasped by our theory, which predicts $L_c^{\mathrm{pred}}(x_0) = 2.30L_c^{\mathrm{hom}}$ (18% error). The proposed framework describes then fairly well the stability behavior of *very heterogeneous* faults. The observed errors might be due to the limits of the second-order theory since it assumes $\sigma_w/\langle W\rangle \lesssim 1$, while $\sigma_w/\langle W\rangle = 8$ for the extremal case.

References

Albertini, G., Karrer, S., Grigoriu, M. D., & Kammer, D. S. (2020). Stochastic properties of static friction. *Journal of the Mechanics and Physics of Solids*, 104242. Retrieved from `http://www.sciencedirect.com/science/article/pii/S0022509620304555` doi: 10.1016/j.jmps.2020.104242

Ampuero, J.-P., Ripperger, J., & Mai, P. M. (2006). Properties of dynamic earthquake

ruptures with heterogeneous stress drop. In *Earthquakes: Radiated energy and the physics of faulting* (pp. 255–261). American Geophysical Union (AGU). Retrieved from https://agupubs.onlinelibrary.wiley.com/doi/abs/10.1029/170GM25

Ben-David, O., & Fineberg, J. (2011). Static friction coefficient is not a material constant. *Physical Review Letters*, *106*(25), 254301. Retrieved from https://link.aps.org/doi/10.1103/PhysRevLett.106.254301 doi: 10.1103/PhysRevLett.106.254301

Breitenfeld, M. S., & Geubelle, P. H. (1998, September). Numerical analysis of dynamic debonding under 2D in-plane and 3D loading. *International Journal of Fracture*, *93*(1-4), 13–38. doi: 10.1023/A:1007535703095

Breitkopf, P., Naceur, H., Rassineux, A., & Villon, P. (2005). Moving least squares response surface approximation: Formulation and metal forming applications. *Computers & Structures*, *83*(17), 1411–1428. Retrieved from http://www.sciencedirect.com/science/article/pii/S0045794905000726 doi: 10.1016/j.compstruc.2004.07.011

Brener, E. A., Aldam, M., Barras, F., Molinari, J.-F., & Bouchbinder, E. (2018). Unstable slip pulses and earthquake nucleation as a nonequilibrium first-order phase transition. *Physical Review Letters*, *121*(23), 234302. Retrieved from https://link.aps.org/doi/10.1103/PhysRevLett.121.234302 (Publisher: American Physical Society) doi: 10.1103/PhysRevLett.121.234302

Cao, X., Bouzat, S., Kolton, A. B., & Rosso, A. (2018). Localization of soft modes at the depinning transition. *Physical Review E*, *97*(2), 022118. Retrieved from https://link.aps.org/doi/10.1103/PhysRevE.97.022118 doi: 10.1103/

PhysRevE.97.022118

Dascalu, C., Ionescu, I. R., & Campillo, M. (2000). Fault finiteness and initiation of dynamic shear instability. *Earth and Planetary Science Letters*, *177*(3), 163–176. Retrieved from http://www.sciencedirect.com/science/article/pii/S0012821X00000558 doi: 10.1016/S0012-821X(00)00055-8

Démery, V., Lecomte, V., & Rosso, A. (2014). The effect of disorder geometry on the critical force in disordered elastic systems. *Journal of Statistical Mechanics: Theory and Experiment*, *2014*(3), P03009. Retrieved from https://doi.org/10.1088%2F1742-5468%2F2014%2F03%2Fp03009 doi: 10.1088/1742-5468/2014/03/P03009

Geubelle, P. H., & Rice, J. R. (1995). A spectral method for three-dimensional elastodynamic fracture problems. *Journal of the Mechanics and Physics of Solids*, *43*(11), 1791–1824. Retrieved from http://www.sciencedirect.com/science/article/pii/002250969500043I doi: 10.1016/0022-5096(95)00043-I

Grigoriu, M. (2002). *Stochastic calculus: Applications in science and engineering.* Birkhäuser Basel. Retrieved from https://www.springer.com/gp/book/9780817642426 doi: 10.1007/978-0-8176-8228-6

Lapusta, N., Rice, J. R., Ben-Zion, Y., & Zheng, G. (2000). Elastodynamic analysis for slow tectonic loading with spontaneous rupture episodes on faults with rate- and state-dependent friction. *Journal of Geophysical Research: Solid Earth*, *105*, 23765–23789. Retrieved from https://agupubs.onlinelibrary.wiley.com/doi/abs/10.1029/2000JB900250 doi: https://doi.org/10.1029/2000JB900250

Latour, S., Schubnel, A., Nielsen, S., Madariaga, R., & Vinciguerra, S. (2013). Charac-

terization of nucleation during laboratory earthquakes. *Geophysical Research Letters*, *40* (19), 5064–5069. Retrieved from https://agupubs.onlinelibrary.wiley.com/doi/abs/10.1002/grl.50974 doi: 10.1002/grl.50974

Perrin, G., & Rice, J. (1994). Disordering of a dynamic planar crack front in a model elastic medium of randomly variable toughness. *Journal of the Mechanics and Physics of Solids*, *42* (6), 1047–1064. Retrieved from http://www.sciencedirect.com/science/article/pii/0022509694900833 doi: 10.1016/0022-5096(94)90083-3

Rubin, A. M., & Ampuero, J.-P. (2005). Earthquake nucleation on (aging) rate and state faults. *Journal of Geophysical Research: Solid Earth*, *110*. Retrieved from https://agupubs.onlinelibrary.wiley.com/doi/abs/10.1029/2005JB003686 doi: 10.1029/2005JB003686

Shinozuka, M., & Deodatis, G. (1991). Simulation of stochastic processes by spectral representation. *Applied Mechanics Reviews*, *44* (4), 191–204. Retrieved from https://asmedigitalcollection.asme.org/appliedmechanicsreviews/article/44/4/191/400800/Simulation-of-Stochastic-Processes-by-Spectral doi: 10.1115/1.3119501

Svetlizky, I., Muñoz, D. P., Radiguet, M., Kammer, D. S., Molinari, J.-F., & Fineberg, J. (2016). Properties of the shear stress peak radiated ahead of rapidly accelerating rupture fronts that mediate frictional slip. *Proceedings of the National Academy of Sciences*, *113* (3), 542–547. Retrieved from https://www.pnas.org/content/113/3/542 doi: 10.1073/pnas.1517545113

Tanguy, A., & Vettorel, T. (2004). From weak to strong pinning i: A finite size study. *The*

European Physical Journal B - Condensed Matter and Complex Systems, *38*(1), 71–82. Retrieved from https://doi.org/10.1140/epjb/e2004-00101-6 doi: 10.1140/epjb/e2004-00101-6

Uenishi, K., & Rice, J. R. (2003). Universal nucleation length for slip-weakening rupture instability under nonuniform fault loading. *Journal of Geophysical Research: Solid Earth*, *108*. Retrieved from https://agupubs.onlinelibrary.wiley.com/doi/abs/10.1029/2001JB001681 doi: 10.1029/2001JB001681

Viesca, R. C. (2016a). Self-similar slip instability on interfaces with rate- and state-dependent friction. *Proceedings of the Royal Society A: Mathematical, Physical and Engineering Sciences*, *472*(2192), 20160254. Retrieved from https://royalsocietypublishing.org/doi/10.1098/rspa.2016.0254 doi: 10.1098/rspa.2016.0254

Viesca, R. C. (2016b). Stable and unstable development of an interfacial sliding instability. *Physical Review E*, *93*(6), 060202(R). Retrieved from https://link.aps.org/doi/10.1103/PhysRevE.93.060202 doi: 10.1103/PhysRevE.93.060202

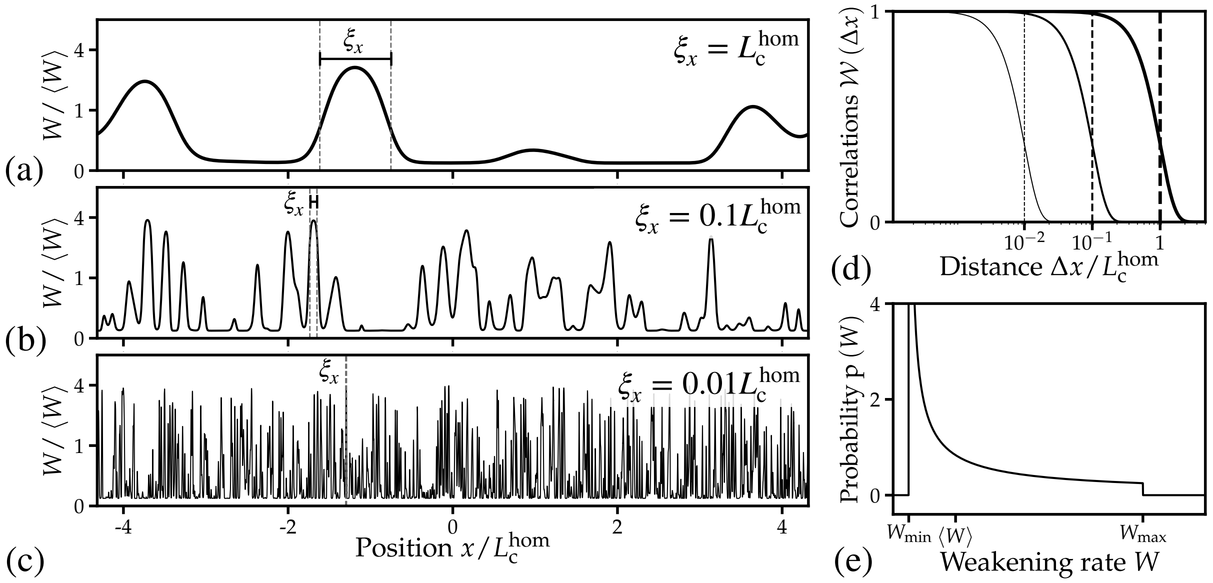

Figure S1. Examples of W field generated by the procedure of (Albertini et al., 2020) for three different correlation lengths: (a) $\xi_x = L_c^{hom}$, (b) $\xi_x = 0.1L_c^{hom}$, and (c) $\xi_x = 0.01L_c^{hom}$. (d) These fields follow Gaussian correlations $\mathcal{W}(|\Delta x|/\xi_x) = e^{-(\Delta x/\xi_x)^2}$, and (e) their values are distributed following a beta-distribution $\mathrm{p}(W)$ between two extremal values $[W_{\min}, W_{\max}]$.

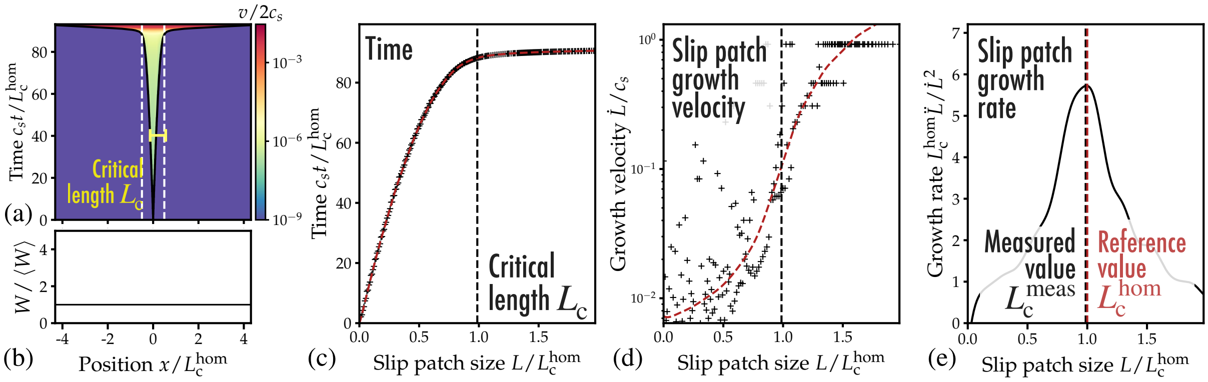

Figure S2. (a) The nucleation dynamics is composed of a first phase of quasi-static growth of the slip patch followed by a phase of dynamic crack propagation when its size L reaches a critical value L_c for (b) a homogeneous fault. This shift of dynamics can be observed on (c) the temporal evolution of the slip patch size $L(t)$. A better option is to measure L_c^{meas} as the point where (d) its growth velocity $\dot{L}$ hits an inflection point (in linear-log), i.e. (e) when the growth rate $\ddot{L}/\dot{L}^2$ is maximal, in accordance with the theoretical solution $L_c^{hom} = 1.158 L_c^{hom}$. Note that $\dot{L}$ and $\ddot{L}/\dot{L}^2$ are estimated from the noisy signal $L(t)$ with a moving weighted least-square procedure.

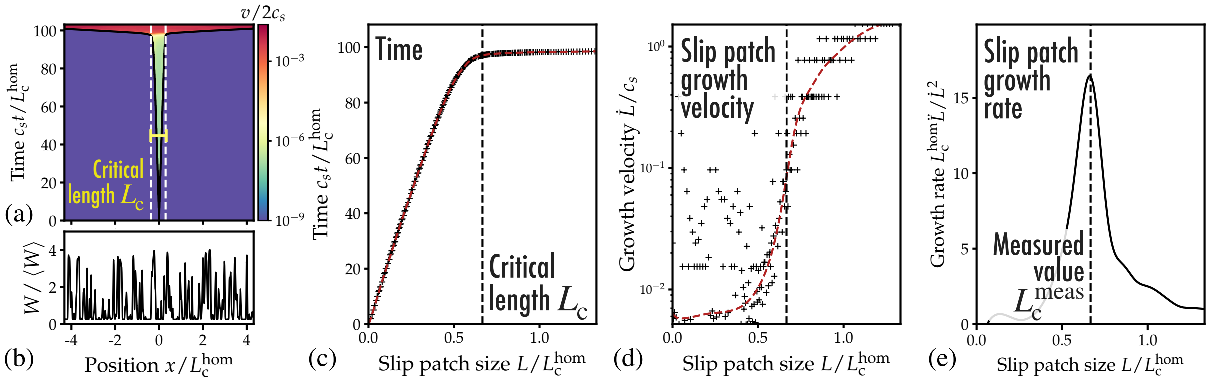

Figure S3. (a) The nucleation dynamics is composed of a first phase of quasi-static growth of the slip patch followed by a phase of dynamic crack propagation when its size L reaches a critical value L_c for (b) a heterogeneous fault where the weakening rate W fluctuates with the position. As in the homogeneous case, the nucleation event can be alternatively observed on (c) the temporal evolution of the slip patch size L, (d) its growth velocity $\dot{L}$, or (e) the growth rate $\ddot{L}/\dot{L}^2$. The latter offers a robust measurement of the critical length L_c^{meas} at a ±5% precision.

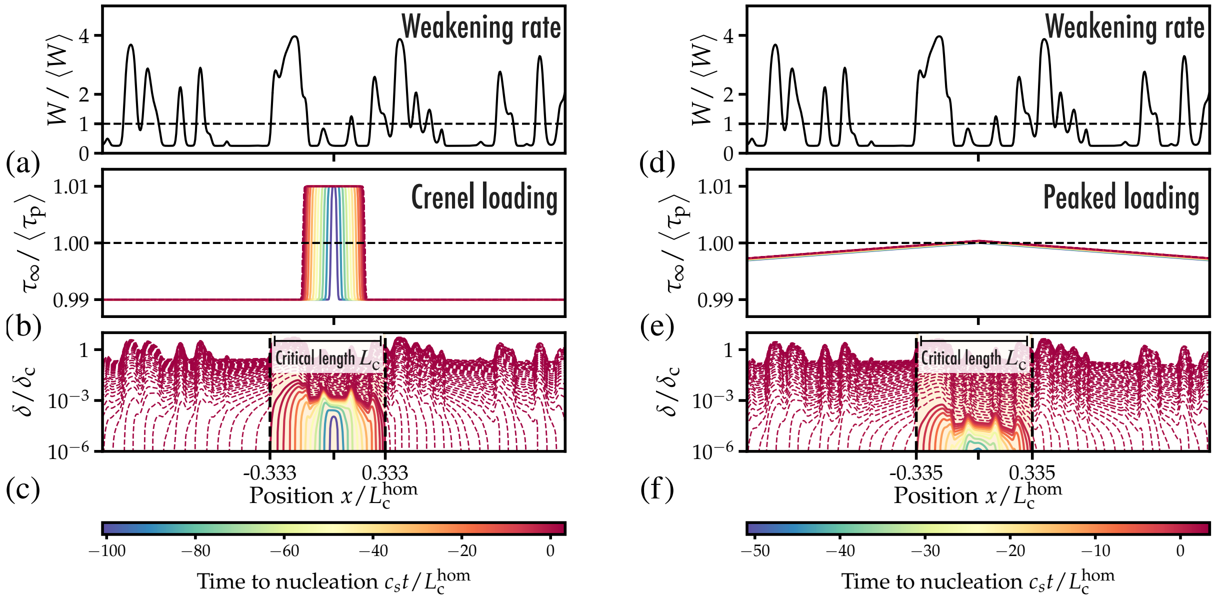

Figure S4. (a) & (d) For the same given disorder realization of weakening rate W, the nucleation dynamics do not depend on the precise loading shape, be it (b) crenel-like or (e) linearly-peaked, as long as it is peaked on the center of the fault to force the nucleation on this part of the interface. In particular, if (c) & (f) the value of the local slip δ might depend on the loading type, the critical length L_c to instability is left unchanged (within the ±5% measurement errors).

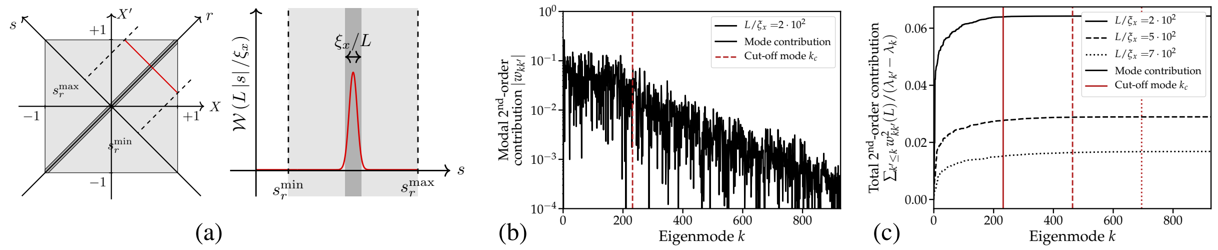

Figure S5. (a) Diagonal domination argument of (Perrin & Rice, 1994): for small ξ_x/L, the region where $\mathcal{W}\left(L\left|s\right|/\xi_x\right)$ takes non-negligible values is "concentrated" near the diagonal of the square of integration $\left[-1,1\right]\times\left[s_r^{\mathrm{min}}, s_r^{\mathrm{max}}\right]$. It implies that (b) $w_{kk'}\left(L\right)^2$ (black solid line) is only taking non-negligeable values up to $k_{\mathrm{c}}\simeq\left\lceil 2L/\xi_x\right\rceil$ (red dashed line). (c) All the second-order modal contributions of Eq. S(26) are then accounted for when summing up to k_{c}.

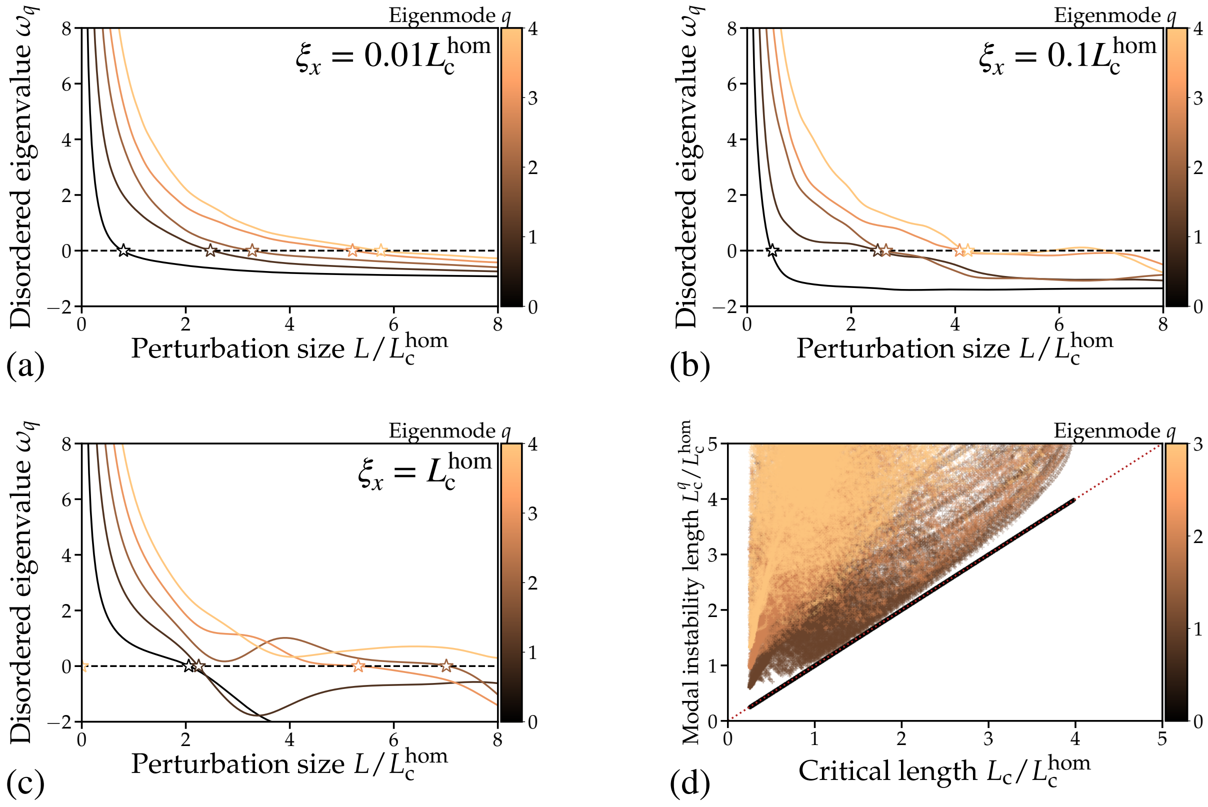

Figure S6. The critical length L_c, defined as the smallest modal instability length L_c^k satisfying $\omega_k\left(L_c^k\right) = 0$, is always reached by the first eigenmode $k = 0$ no matter the characteristics of the disorder: (a) $\xi_x = 0.01L_c^{hom}$, (a) $\xi_x = 0.1L_c^{hom}$, and (c) $\xi_x = L_c^{hom}$, despite being sometimes close. (d) This observation is in accordance with the measurement of $\left(L_c^k\right)_{k\in[0,3]}$ using Eq. S(26) for 45'000 nucleation events.

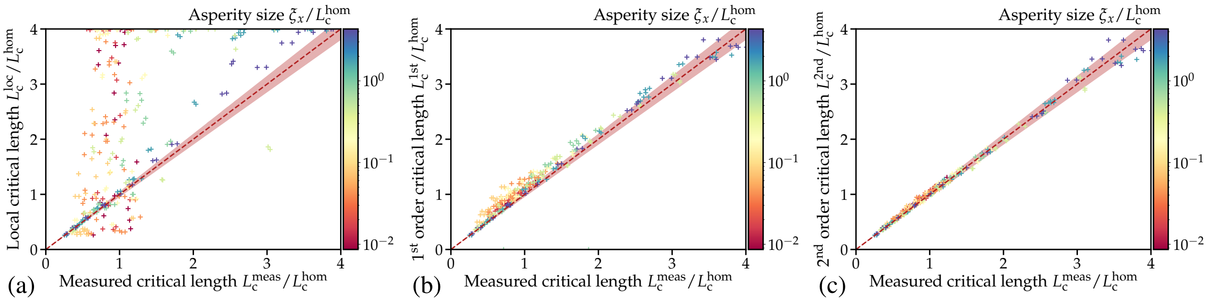

Figure S7. The length of the critical nucleus L_c^{meas} measured from the numerical dynamic calculations (a) cannot be inferred from the local critical length $L_c^{loc} \simeq 1.158\mu^*/W(0)$ that corresponds to a purely individual behavior. Eq. (4) takes into account the collective depinning of the local asperities that ultimately leads to interfacial failure. (b) First-order terms (two first terms of Eq. (4)) significantly improves the prediction of the critical length L_c, but (c) second-order terms (third term of Eq. (4)) are required to achieve a good agreement.

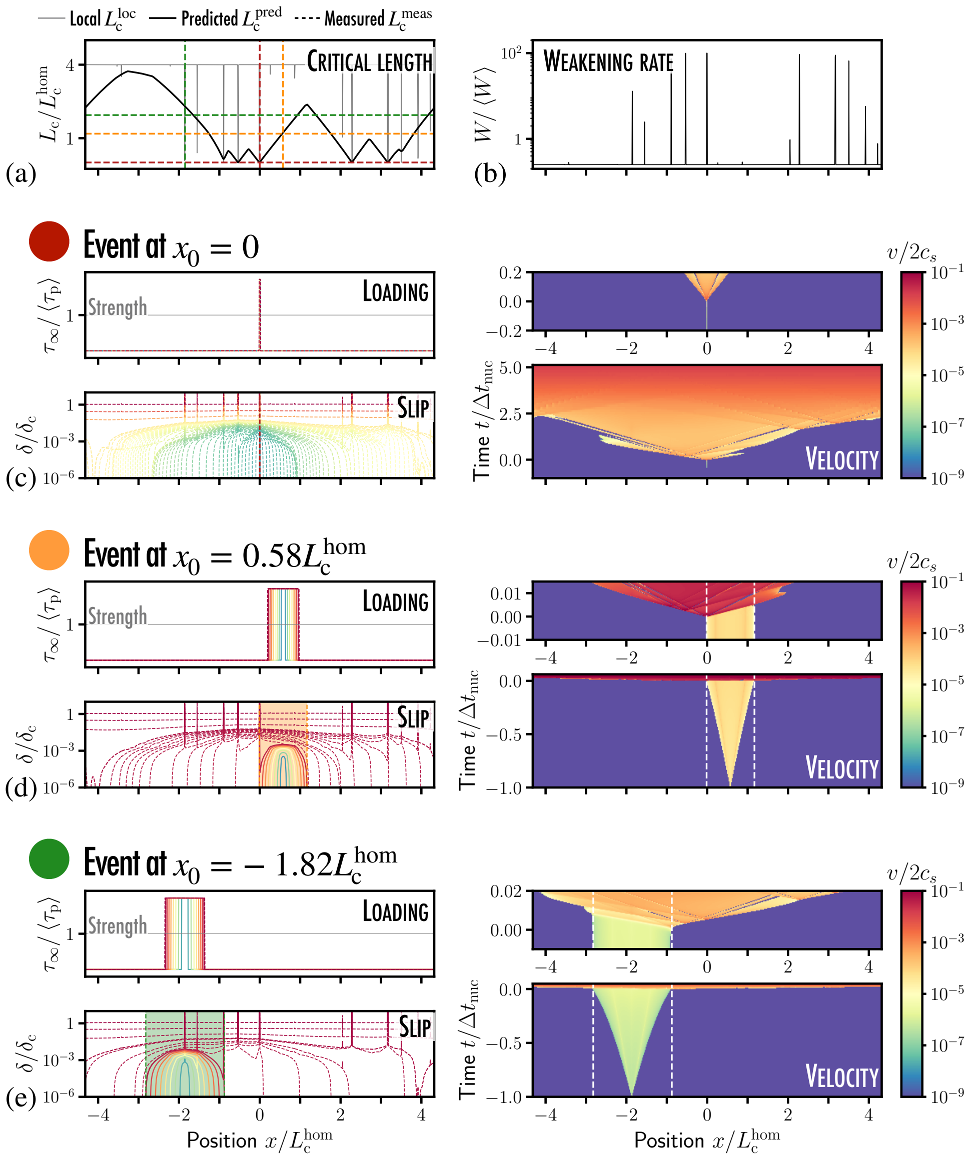

Figure S8. Focus on the extremal regime of Figure 3e (main text): (a) the effective nucleation length L_c^{pred}, which is computed with Eq. (4) from (b) the spatial variations of the weakening rate W, display strong variations along the fault axis. The dashed colored lines corresponds to the position and size of the nucleation measured numerically for each event considered (c-e). (c) (Red event) At $x_0 = 0$, the fault stability is governed by a critical defect of size $\xi_x = 0.02L_c^{hom}$ in which an instability of critical size $L_c^{min} = 0.01L_c^{hom}$ nucleates. (d) (Orange event) At $x_0 \simeq 0.58L_c^{hom}$, the nucleation length increases to $L_c^{meas} = 1.18L_c^{hom}$ and is mainly controlled by the distance to a critical defect. (e) (Green event) At $x_0 \simeq -1.82L_c^{hom}$, the nucleation length is still large $L_c^{meas} = 1.96L_c^{hom}$, even if the nucleation is forced on a weak location where the local critical length drops to $L_c^{loc}(x_0) \simeq 0.08L_c^{hom}$.
